# Utility of Continuous Disease Subtyping Systems for Improved Evaluation of Etiologic Heterogeneity

**DOI:** 10.3390/cancers14071811

**Published:** 2022-04-02

**Authors:** Ruitong Li, Tomotaka Ugai, Lantian Xu, David Zucker, Shuji Ogino, Molin Wang

**Affiliations:** 1 Broad Institute of MIT and Harvard, Cambridge, MA 02142, USA; ruitong@broadinstitute.org (R.L.); sogino@bwh.harvard.edu (S.O.); 2 Department of Epidemiology, Harvard T.H. Chan School of Public Health, Boston, MA 02115, USA; tugai@bwh.harvard.edu; 3 Program in MPE Molecular Pathological Epidemiology, Department of Pathology, Brigham and Women’s Hospital, Harvard Medical School, Boston, MA 02115, USA; 4 Department of Biostatistics, Harvard T.H. Chan School of Public Health, Boston, MA 02115, USA; lxu@hsph.harvard.edu; 5 Department of Statistics and Data Science, Hebrew University, Jerusalem 91905, Israel; david.zucker@mail.huji.ac.il; 6 Cancer Immunology and Cancer Epidemiology Programs, Dana-Farber Harvard Cancer Center, Boston, MA 02115, USA; 7 Channing Division of Network Medicine, Department of Medicine, Brigham and Women’s Hospital, Harvard Medical School, Boston, MA 02115, USA

**Keywords:** bioinformatics, environment, epigenomics, immune response, immunology, interdisciplinary research, microbiology, molecular epidemiology, targeted intervention, time-to-event data

## Abstract

**Simple Summary:**

This paper presents an extended version of the Cox regression model to examine heterogeneous effects of risk factors on disease subtypes defined by a continuous biomarker. This approach can be easily applied to cancer studies and is accessible to researchers via user-friendly R scripts.

**Abstract:**

Molecular pathologic diagnosis is important in clinical (oncology) practice. Integration of molecular pathology into epidemiological methods (i.e., molecular pathological epidemiology) allows for investigating the distinct etiology of disease subtypes based on biomarker analyses, thereby contributing to precision medicine and prevention. However, existing approaches for investigating etiological heterogeneity deal with categorical subtypes. We aimed to fully leverage continuous measures available in most biomarker readouts (gene/protein expression levels, signaling pathway activation, immune cell counts, microbiome/microbial abundance in tumor microenvironment, etc.). We present a cause-specific Cox proportional hazards regression model for evaluating how the exposure–disease subtype association changes across continuous subtyping biomarker levels. Utilizing two longitudinal observational prospective cohort studies, we investigated how the association of alcohol intake (a risk factor) with colorectal cancer incidence differed across the continuous values of tumor epigenetic DNA methylation at long interspersed nucleotide element-1 (LINE-1). The heterogeneous alcohol effect was modeled using different functions of the LINE-1 marker to demonstrate the method’s flexibility. This real-world proof-of-principle computational application demonstrates how the new method enables visualizing the trend of the exposure effect over continuous marker levels. The utilization of continuous biomarker data without categorization for investigating etiological heterogeneity can advance our understanding of biological and pathogenic mechanisms.

## 1. Introduction

In clinical medicine, patients who share common symptoms and disease characteristics are grouped into a certain disease entity. However, molecular pathological diagnosis is a part of routine clinical practice, especially in oncology. Pathogenic mechanisms commonly vary between patients with the same disease entity. Therefore, when appropriate, patients with the disease are subclassified into groups (disease subtypes) based on their molecular pathological diagnosis to improve clinical management and treatment outcomes. Different disease subtypes are regarded as developing through distinct pathological mechanisms, on which risk factors may exert differential influence [1,2,3,4]. Therefore, the disease-subtyping framework and associated etiological heterogeneity have been widely applied in analyses of both neoplastic and non-neoplastic diseases [5,6,7]. For example, subtype heterogeneity has been identified when investigating the specific effects of a polygenic risk score and breastfeeding for breast cancer subtypes: basal-like and ERBB2 (HGNC ID: 3430; so-called HER2)-overexpressing breast cancer [8].

Despite continuous measurement readouts of many biomarkers used for disease subtyping, such continuous biomarker measures are commonly reduced to a small number of categorical levels (sometimes only two or three) to define disease subtypes, which can simplify the statistical analysis and generate readily interpretable data. Therefore, most existing statistical methods for studying etiological heterogeneity have focused on categorical disease subtype settings [9]. However, this categorization leads to reduction of information in the biomarker data, and is prone to bias due to arbitrary selection of cutoff values. For example, a weakness of categorical subtyping is evident when the exposure effect is limited to patients corresponding to extreme ends of the biomarker measures. In such situations, the patients associated with the exposure effect will likely be submerged among other patients not associated with the exposure effect. As a result, analysis using limited disease subtype categories may fail to discover existing exposure–disease associations. To maximize the value of disease subtyping biomarker information, this article presents an analytical framework for assessing the heterogeneity of exposure–disease subtype associations using continuous biomarker measures instead of categorical subtyping [10].

For illustration, we applied the proposed method to assess how the association of alcohol intake with colorectal cancer incidence changes across DNA methylation level at long interspersed nucleotide element-1 (LINE-1), measured in tumors. We used data from two prospective cohort studies, the Nurses’ Health Study (NHS) and Health Professionals Follow-up Study (HPFS).

## 2. Materials and Methods

To evaluate the association of an exposure with an incident disease in a cohort study, researchers typically use the Cox model [11], in which the hazard function is modeled as

(1) $$\lambda \left(t\;|\;X_{i}\left(t\right),\;W_{i}\left(t\right)\right)=\lambda_{0}\left(t\right)exp\left\{\beta X_{i}\left(t\right)+\gamma^{T\;}W_{i}\left(t\right)\right\}$$

where $\lambda_{0}\left(t\right)$ is the baseline hazard at time t, $X_{i}$ is the possibly time-varying exposure for the *i*-th individual, the coefficient β of X, represents the exposure–outcome association, $W_{i}$ is a p×1 vector of potential confounders, which may also be time-varying, for the *i*-th individual, and γ is a p×1 vector of regression coefficients for W. Without further specification, we assumed that the exposure is a scalar throughout this paper for notational simplicity.

Now, it is of interest to evaluate how the association of an exposure with the disease risk changes over the level of a disease marker. Extending Equation (1), we model the cause-specific hazards [12] of the disease subtypes by incorporating a function of the marker’s value as the coefficient of the exposure. Our model is

(2) $$\lambda_{z}\left(t\;|\;X_{i}\left(t\right),\;W_{i}\left(t\right)\right)=\lambda_{z0}\left(t\right)exp\left\{g\left(\phi ,Z\right)X_{i}\left(t\right)+\gamma^{T\;}W_{i}\left(t\right)\right\}$$

where Z is the continuous disease marker (cause), $\lambda_{z0}\left(t\right)$ and $\lambda_{z}\left(t\right)$ are the baseline hazard and hazard functions for disease with marker level Z, and g(ϕ,Z) is a given real-valued function of Z with unknown parameters ϕ. The association between the exposure and the disease with marker level Z can be then represented by the hazard ratio HR(Z)=exp{g(ϕ,Z)}. If the exposure is a q-dimensional column vector, its coefficient will also be vector-valued with the form $\left(g_{1}\left(\phi^{\left(1\right)},Z\right),g_{2}\left(\phi^{\left(2\right)},\;Z\right),\ldots ,g_{q}\left(\phi^{\left(q\right)},\;Z\right)\right)$, where $g_{k}$ is the function of the disease marker corresponding to the coefficient of the *k*-th element of the exposure, and $\phi^{\left(q\right)}$ is a scalar or vector parameter of interest, k=1,…,q.

The regression coefficients in the standard Cox model (1) are typically estimated by maximizing the partial likelihood [13]. Under the cause-specific proportional hazards model (2), we can construct the corresponding partial likelihood [14] as follows:

(3) $$PL=\underset{i\in C}{\prod }\frac{exp\;\left\{g\left(\phi ,Z_{i}\right)X_{i}\left(T_{i}\right)+\gamma^{T\;}W_{i}\left(T_{i}\right)\right\}\;}{\sum_{l}I\left(T_{l}\geq T_{i}\right)exp\left\{g\left(\phi ,Z_{i}\right)X_{l}\left(T_{i}\right)+\gamma^{T\;}W_{l}\left(T_{i}\right)\right\}}$$

where C is the set of all cases and T is the time to event, which in a cohort study is typically age at disease diagnosis. Statistical software for the standard Cox model does not work here, as the marker level Z in g(ϕ,Z) is defined only among cases. In this partial likelihood, the subjects in a risk set are assigned the marker value of the case in that risk set so that the numerator and denominator in PL correspond to the hazard defined at the same marker level. The parameters ϕ and γ in Model (2) can be estimated through maximizing this partial likelihood. Similar to the standard Cox model setting, the variances of the parameter estimates can be estimated based on the inverse of the Hessian matrix.

We suggest using the restricted cubic spline approach [15] to characterize g(ϕ,Z). The restricted cubic spline approach has advantages of parsimony while allowing for great flexibility in characterizing nonlinear curves. A restricted cubic spline function g(ϕ,Z) with K (≥3) knots includes one intercept, one linear, and K−2 nonlinear terms of *Z*; that is,

(4) $$g\left(\phi ,Z\right)=\phi_{0}+\phi_{1}Z+\sum_{j=1}^{K-2}\phi_{j+1}S_{j}\left(Z\right),$$

where $S_{j}\left(Z\right)\;$ is the *j*-th basis function of the restricted cubic spline, evaluated at *Z*. See Supplementary Material Section S1 for details. If K=2, g(ϕ,Z) only includes the intercept and the linear term. The unknown parameter ϕ contains the intercept and all the coefficients of the linear and nonlinear terms. The number of knots can be determined using the Akaike information criterion (AIC) or the Bayesian information criterion (BIC) [16], and typically, the knots can be evenly spaced over the distribution of Z.

We used the likelihood ratio test to test for zero elements of ϕ. All elements of ϕ being zero implies no exposure–outcome association. Non-zero intercept and zero coefficients of all the linear and nonlinear terms imply an exposure–disease association that is independent of the disease marker. A non-zero coefficient of the linear term along with zero coefficients of all the nonlinear terms implies that the exposure–outcome association increases or decreases linearly over the marker level.

## 3. Results

### 3.1. Simulation Study

We conducted a simulation study to assess the finite sample performance of the method when K=3. See Supplementary Material Section S2 for details. This simulation study shows that the point estimate $\hat{\phi }$ of ϕ performs satisfactorily (Table S1 in the Supplementary Material Section S2). When the number of cases was 900, the percent bias of $\hat{\phi }$ was 4 to 8% in five out of six configurations and 11% in the last configuration. It was 0.3 to 4% in five out of six configurations and 9.7% in the last configuration when the number of cases was increased to 4500. The empirical standard error of $\hat{\phi }$ decreased by about 60% when the number of cases were increased from 900 to 4500.

### 3.2. Results of Illustrative Example

We used colorectal cancer (adenocarcinoma) and its subtyping biomarker, LINE-1 methylation (with continuous unitless values) [17], as a disease biomarker example to illustrate the method. We utilized data from ongoing large prospective cohort studies, namely the Nurses’ Health Study (NHS) [18,19] and Health Professionals Follow-up Study (HPFS) [20,21]. The main exposure was cumulative average alcohol intake (0, ≤15, >15 g/day). Detailed descriptions of the study population, assessment of main exposure and covariates, ascertainment of colorectal cancer cases, and quantification of LINE-1 levels are described in Supplementary Material Section S3. The age-standardized characteristics of participants in the two cohorts are summarized in Table S2 (Supplementary Material).

**Figure 1:**
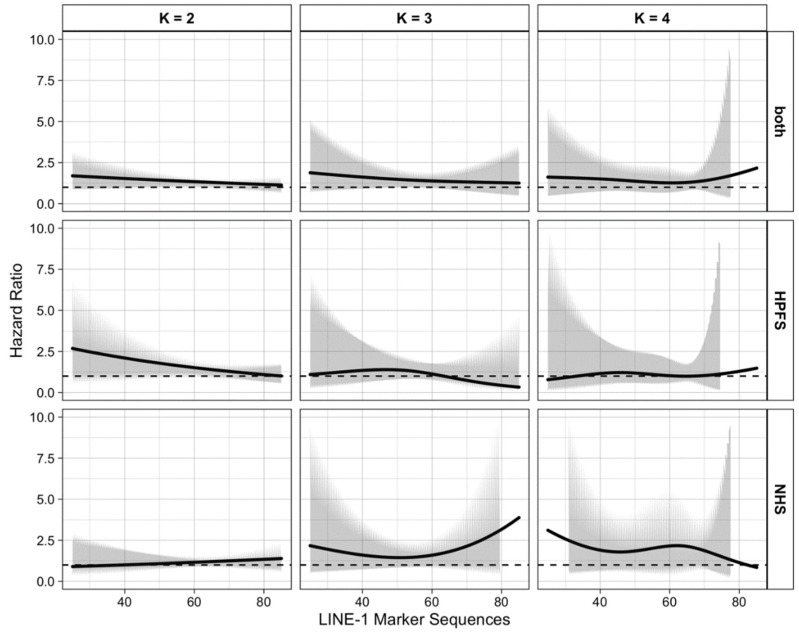
Heterogeneous Effect of Cumulative Categorical Alcohol Intake (>15 g/day vs. 0 g/day) on continuous subtypes of colorectal cancer; the 3 × 3 plot panel illustrates the combination of three choices of the knot number in g(ϕ,Z)  and three cohort settings. Abbreviations: HPFS, Health Professionals Follow-up Study; LINE-1, long interspersed nucleotide element-1; NHS, Nurses’ Health Study.

Shown in Figure 1 and Figure S1 (Supplementary Material) are the curves of the hazard ratios (HRs) representing the association between alcohol intake and incidence of colorectal cancer subtype as a function of continuous LINE-1 methylation level. These curves were constructed by plotting $exp\left\{g\left(\hat{\phi },Z\right)\right\}$ over the LINE-1 marker values (Z) within the plausible range (25 to 85). The number of knots considered were K=2, 3, 4. The knots were evenly spaced over the LINE-1 distribution. Figure 1 and Figure S1 were drawn based on the results using the combined cohort, HPFS alone, and NHS alone. We considered two models: the main model, with stratification factors only, and the full model, which adjusted for additional covariates as described in the Methods section. Since the inclusion of additional covariates in the full model had little impact on the set of estimated coefficients ϕ, we simply utilized the estimation results from the main model hereafter.

Table 1 and Table S3 (Supplementary Material) present *p*-values from testing the following null hypotheses for the same choices of knot numbers and cohort settings as in Figure 1 and Figure S1: (i) the intercept and all the coefficients in g(ϕ,Z) are zero (the overall test); (ii) all the coefficients in g(ϕ,Z) except the intercept are zero (test for heterogeneity); (iii) all the coefficients of the nonlinear terms in g(ϕ,Z) are zero (test for nonlinearity). For the NHS cohort and the combined cohort, the linear model (K=2) had the smallest BIC and AIC, and for the HPFS cohort, the linear model had the smallest BIC and the model with K=3 had the smallest AIC. For the comparison between >15 g/day intake and 0 g/day based on the models with K=2, 3, as shown in Table 1, there were significant associations between alcohol and cancer risk in the HPFS cohort (overall test *p* < 0.001) and the combined cohort (overall test *p* < 0.001), but there was insufficient statistical evidence to establish such an association in the NHS cohort. There was insufficient statistical evidence to establish a difference in the comparison of ≤15 g/day intake versus 0 g/day in the NHS, HPFS, or the combined cohort (Table S3). Furthermore, in the comparison of >15 g/day versus 0 g/day in the combined cohort, the heterogeneity tests were statistically significant (*p* < 0.001) under K=2, 3, and the alcohol effect changed with the LINE-1 level linearly (nonlinear test *p* = 0.54 for K=3).

Table 2 and Table S4 (Supplementary Material) display the estimated HRs, with 95% pointwise confidence intervals, representing the alcohol–cancer association for some plausible LINE-1 values (30 to 80 in steps of 10) for the choices of knot numbers and data settings considered in Figure 1. As shown for the HPFS and combined cohorts in Figure 1 and Table 2, the alcohol–cancer association (for >15 g/day vs. 0 g/day) tended to decrease with increasing LINE-1 methylation level, as seen from the two g(ϕ,Z) functions with K=2, 3 as selected by AIC and BIC.

## 4. Discussion

In this paper, we have presented a Cox proportional hazards regression model method to fully utilize a continuous biomarker measure for disease subtyping. This statistical method can examine subtype heterogeneity of diseases in the exposure–disease association with more comprehensive and versatile utilization of continuous marker measurements. The ability of this method to potentially reveal more complicated patterns in subtype heterogeneity can help us gain deeper insights into etiologies in molecular epidemiological research and provide further evidence in the development of personalized precision medicine.

Statistical methods for investigating disease subtype heterogeneity for categorical and ordinal subtypes have been studied previously under several common study designs [9]. However, a concern may be raised about defining discrete subtypes based on categorization of biomarker values when there is little or no evidence supporting biomarker cut-point values that are often arbitrarily determined. In addition, the categorization of a continuous measure of a biomarker can lead to loss of information from the biological and statistical perspectives. The proposed method is less prone to these problems and has the potential to reveal more detailed and granular subtype heterogeneity than established approaches using categorical and ordinal subtypes.

Many biological phenomena and related biomarkers (including expressions of genes and proteins) are continuous in nature [6]. LINE-1 methylation level (i.e., the percentage of the amount of C nucleotides divided by the sum of the amounts of C and T nucleotides at CpG sites), which we used in the illustrative example, is a surrogate marker for genome-wide DNA methylation and widely distributed in colorectal cancer tissue from 20 to 90% [22,23]. Currently, it remains unclear how to set the best cut-points for defining subtypes based on quantitative LINE-1 methylation levels. Accordingly, the proposed method can be applied to this biomarker without using arbitrarily cut-points. Another example for continuous tissue biomarkers is immune cell infiltrates in tumor tissue. Ample evidence supports the biological importance of the immune system in cancer [24,25,26,27]. Tumors exhibit considerably heterogeneous phenotypes according to types and quantities of immune cell infiltrates in tumor tissue [28,29], and higher immune cell infiltrates in cancer have often been associated with better cancer survival [26,30,31,32]. Related to immune cells, microbial species are often quantitatively measured in biospecimens including tumor and normal tissue in population studies [33,34]. Readouts of quantitative microbial assays are continuous in nature without prior knowledge on any biological cut-points (or threshold effect). Categorizations of such variables are often used [35,36,37,38]. However, simple categorizations may lose biological information. It is evident that standardized definitions of tumor subtypes based on immune cell infiltrates or tissue microbiota have not been developed. There is a clear need to analyze tumor biomarker data in a way that exploits the underlying continuous nature of the biomarker.

The real-world application of this method in the two large prospective cohort studies has demonstrated its capability to depict the trend of the exposure effect across continuous molecular marker levels in contrast to use of solely categorical subtypes [10]. Further, this method allows for the flexible modeling of the heterogeneous effect of exposure on the disease of interest across biomarker levels, using models ranging from linear functions, to functions of any hypothesized form, to a case-by-case understanding of the disease.

A user-friendly R program that implements this method is publicly available (https://www.hsph.harvard.edu/molin-wang/software/, accessed on 31 March 2022). This R function fits a Cox regression model for either incidence analysis or post-diagnosis survival analysis, where the model can include one or more exposure variables, a set of confounders (optional), and one or more stratification variables (optional). Left truncation and time-varying covariates, which are common in cohort data analyses, can be handled by putting the data in counting process form [39] before applying our R function. In the counting process data structure, a new data record is created for each questionnaire cycle at which a participant was at risk, with covariates set to their values at the time the questionnaire was returned. Furthermore, in addition to AIC and BIC, the cross validation approach [40] could also be used to choose the number of knots in the restricted cubic spline approach. The proposed method can be easily applied to studies of various diseases and risk factors and is accessible to researchers with limited experience with time-to-event data analysis.

In this article, we follow the nomenclature guideline for gene products using the Human Genome Organization (HUGO) Gene Nomenclature Committee (HGNC) standards, recommended by the expert panel [41].

## 5. Conclusions

To summarize, we have presented a Cox proportional hazards regression model for analyzing heterogeneous exposure–disease associations across disease subtypes defined by continuous biomarker measures. This method is helpful in decreasing bias caused by arbitrary subtype categorization and in increasing statistical power, as well as flexibility of assumptions about the pattern of pathologic heterogeneity. The utilization of continuous marker data without categorization for investigating subtype heterogeneity will advance our understanding of etiological heterogeneity and possibly contribute to precision medicine.

## Figures and Tables

**Table 1:** Model testing for the association of categorical alcohol intake (>15 g/day vs. 0 g/day) with colorectal cancer incidence, based on the main model for three functional forms and three cohorts.

Knots	Model Assessment	NHS	HPFS	Combined
*K* = 2	*p*-value			
	Overall	0.19	<0.001	<0.001
	Heterogeneity	-	<0.001	<0.001
	BIC	11,634	7784	20,436
	AIC	11,586	7739	20,386
*K* = 3	*p*-value			
	Overall	0.12	<0.001	<0.001
	Heterogeneity	-	<0.001	<0.001
	Nonlinearity	-	<0.001	0.54
	BIC	11,660	7804	20,464
	AIC	11,588	7736	20,389
*K* = 4	*p*-value			
	Overall	0.17	<0.001	0.002
	Heterogeneity	-	<0.001	<0.001
	Nonlinearity	-	<0.001	0.56
	BIC	11,686	7830	20,492
	AIC	11,589	7741	20,393

All *p*-values reported above are two sided. Hypothesis testing: H_0_: the intercept and all the coefficients in g(ϕ,Z) are zero (the overall test); H_0_: all the coefficients in g(ϕ,Z) except the intercept are zero (test for heterogeneity); H_0_: all the coefficients of the nonlinear terms in g(ϕ,Z) are zero (test for nonlinearity). Abbreviations: AIC, Akaike’s information criterion; BIC, Bayesian information criterion; HPFS, Health Professionals Follow-up Study; LINE-1, long interspersed nucleotide element-1; NHS, Nurses’ Health Study.

**Table 2:** Hazard ratio for categorical alcohol intake (>15 g/day vs. 0 g/day) modeled using three functional forms for the LINE-1 marker value in three cohort settings, based on the main model.

Cohort	LINE-1 Methylation Level	Hazard Ratio with 95% Confidence Interval					
		Linear Function (K = 2)		Restricted Cubic Spline (K = 3 Knots)		Restricted Cubic Spline (K = 4 Knots)	
Combined	30	1.64	(0.95, 2.82)	1.79	(0.76, 4.21)	1.59	(0.55, 4.61)
	40	1.53	(1.03, 2.28)	1.62	(0.91, 2.87)	1.51	(0.76, 3.01)
	50	1.43	(1.10, 1.86)	1.48	(1.03, 2.13)	1.37	(0.78, 2.40)
	60	1.34	(1.14, 1.58)	1.38	(1.00, 1.92)	1.27	(0.72, 2.22)
	70	1.25	(1.05, 1.50)	1.32	(0.77, 2.26)	1.40	(0.74, 2.64)
	80	1.17	(0.87, 1.57)	1.28	(0.53, 3.05)	1.85	(0.18, 18.5)
HPFS	30	2.47	(1.12, 5.48)	1.18	(0.32, 4.32)	0.91	(0.17, 4.87)
	40	2.10	(1.18, 3.75)	1.35	(0.57, 3.16)	1.16	(0.40, 3.31)
	50	1.78	(1.22, 2.61)	1.38	(0.82, 2.32)	1.18	(0.54, 2.59)
	60	1.52	(1.19, 1.93)	1.13	(0.72, 1.77)	1.02	(0.52, 2.00)
	70	1.29	(0.98, 1.70)	0.74	(0.35, 1.56)	1.03	(0.33, 3.22)
	80	1.09	(0.7, 1.710)	0.43	(0.13, 1.50)	1.29	(0.03, 63.8)
NHS	30	0.94	(0.41, 2.15)	1.95	(0.56, 6.82)	2.58	(0.61, 10.9)
	40	1.01	(0.54, 1.86)	1.60	(0.69, 3.73)	1.90	(0.74, 4.91)
	50	1.08	(0.72, 1.63)	1.45	(0.84, 2.50)	1.85	(0.82, 4.17)
	60	1.16	(0.90, 1.49)	1.59	(0.98, 2.58)	2.16	(0.86, 5.43)
	70	1.25	(0.97, 1.61)	2.14	(1.00, 4.57)	1.87	(0.91, 3.86)
	80	1.34	(0.89, 2.03)	3.17	(0.94, 10.7)	1.14	(0.08, 15.6)

Abbreviations: HPFS, Health Professionals Follow-up Study; LINE-1, long interspersed nucleotide element-1; NHS, Nurses’ Health Study.

## Data Availability

The datasets generated and/or analyzed during the current study are not publicly available. Further information including the procedures to obtain and access data from the Nurses’ Health Studies and the Health Professionals Follow-up Study is described at https://www.nurseshealthstudy.org/researchers/, accessed on 1 February 2022 and https://sites.sph.harvard.edu/hpfs/for-collaborators/, accessed on 1 February 2022.

## Supplementary Materials

The following are available online at https://www.mdpi.com/article/10.3390/cancers14071811/s1, Table S1: Simulation Results for K = 3; 10% censoring rate; 1000 simulation replicates, Table S2: Age-Standardized Characteristics for Study Participants in the NHS (1980–2012) and the HPFS (1986–2012), Table S3: Model Testing for the Association of Alcohol Intake (≤15 g/day Versus 0 g/day) with Colorectal Cancer Incidence, Based on Main Model in Three Functions and Three Settings, Table S4: Hazard Ratio for Alcohol Intake (≤15 g/day Versus 0 g/day) Modeled as Three Functions of LINE-1 Marker Value in Three Cohort Settings, Based on the Main Model. Figure S1: Heterogeneous Effect of Cumulative Categorical Alcohol Intake (≤15 g/day Versus <0 g/day) on Continuous Subtypes of Colorectal Cancer; the 3 × 3 plot panel illustrates the combination of three choices of the knot number in g(ϕ,Z) and three cohort settings.

## Abbreviations

AIC	Akaike information criterion
BIC	Bayesian information criterion
HPFS	Health Professionals Follow-up Study
HR	hazard ratio
LINE-1	long interspersed nucleotide element-1
NHS	Nurses’ Health Study
