# Supplementary material for: Utility of Continuous Disease Subtyping Systems for Improved Evaluation of Etiologic Heterogeneity
Source: Cancers (Basel). 2022 Apr 2;14(7):1811. doi: 10.3390/cancers14071811 (PMC8997600; doi:10.3390/cancers14071811)
Supplement: Supplementary file 1 [file cancers-14-01811-s001.zip › cancers-1638613-supplementary.pdf]

cancers

MDPI

Supplementary Materials

Utility of Continuous Disease Subtyping Systems for Improved Evaluation of Etiologic Heterogeneity

Ruitong Li, Tomotaka Ugai, Lantian Xu, David Zucker, Shuji Ogino and Molin Wang

1. Restricted Cubic Spline Base Functions

Let the knots be $t_1 < t_2 < \cdots < t_k$. The restricted cubic spline base functions are

$$S_j(Z_i) = \left(Z_i - t_j\right)_+^3 - (Z_i - t_{K-1})_+^3(t_k - t_j)/(t_k - t_{K-1}) + (Z_i - t_K)_+^3(t_{k-1} - t_j)/(t_k - t_{K-1}),\ j = 1, \dots, K-2,$$

where $u_+ = u$ if $u > 0$ and $u_+ = 0$ if $u \leq 0$.

2. Details of a Simulation Study

In the simulation study, we assumed there was a binary exposure in the cause-specific hazard model and marker values (Z's) were from the set {0.01, 0.02,…,0.99, 1}. Time to event data were generated based on the exponential distribution and a competing risk framework, where each marker value was treated as a competing event.
The simulation study results are presented in Table S1. We used a small censoring rate (10%) to reduce the computing time. The result should be similar if the censoring rate is larger.

Table S1. Simulation Results for K = 3; 10% censoring rate; 1000 simulation replicates.

True ϕ	Sample Size	%bias ($\hat{\phi}$)	ESE ($\hat{\phi}$)
(0.5, 0.8, 1.1)	1000	(0.043, −0.056, 0.052)	(0.48, 0.92, 0.78)
	5000	(0.028, −0.024, 0.015)	(0.18, 0.38, 0.35)
(−1.5, 0.6, −0.3)	1000	(0.011, 0.076, 0.11)	(0.17, 1.28, 2.98)
	5000	(−0.0034, −0.042, −0.097)	(0.068, 0.48, 1.08)

%bias ($\hat{\phi}$) is $(\hat{\phi} - \phi)/\phi$, averaged over simulation replicates; ESE, the empirical standard error, is the empirical standard deviation over simulation replicates.

3. Materials and Method of Illustrative Examples

3.1. Study Population

The Nurses' Health Study (NHS) and Health Professionals Follow-up Study (HPFS) are two ongoing large prospective studies. Details of both studies have been previously described [22,23]. Briefly, 121 701 U.S. registered female nurses aged between 30 to 55 years were recruited into the NHS in 1976, and 51 529 U.S. male health professionals who were aged 40–75 years were recruited into the HPFS in 1986. The single-gender nature of NHS and HPFS made the sex-stratified investigation of disease risk factors relatively straightforward. The follow-up durations were 1980 to 2012 for the NHS and 1986 to 2012 for the HPFS for this analysis.

3.2. Alcohol Intake and Other Covariates

Detailed information on assessment of alcohol intake and other covariates was described elsewhere [22]. Briefly, information on alcohol intake and other covariates was assessed from the baseline and biennial follow-up questionnaires in both cohorts. To capture long-term exposure, we calculated the cumulative average of alcohol, folate, calcium, red meat intake, body mass index and physical activity, from all preceding questionnaires up to the cycle of interest. For other variables, we used the most recent questionnaire data. Participants were defined as having a positive family history of colorectal cancer if at least one of their parents and siblings had been diagnosed with colorectal cancer.

The Cox proportional hazards regression model analysis for each cohort was based on the counting process data structure and were stratified by age and calendar year of the questionnaire cycle. Additional stratification of sex

was performed for combined analysis of two cohorts. We further adjusted for smoking status (never, ever), body mass index (<25, 25 to 30, or ≥30 kg/m^2), physical activity in unit metabolic equivalent task score (METS)-hour/week (quantile), family history of colorectal cancer in any first-degree relative (yes, no), regular aspirin usage (yes or no), history of colon polyps (yes or no), multi-vitamin usage (yes or no), folate intake (μg/day, quantile), calcium intake (mg/day, quantile) and red meat intake (servings/day, quantile). We used 'main model' to represent the model with solely the stratification variables but not covariates, and 'full model' for the model including the additional covariates.

3.3. Ascertainment of Colorectal Cancer Cases

Incident colorectal cancer cases were ascertained by biennial follow-up questionnaires, the National Death Index (for lethal cases) and medical record review. Study physicians, who were blinded to exposure data, reviewed medical records of identified colorectal carcinoma cases to confirm the pathological diagnosis and extract data on clinical characteristics.

3.4. Quantification of LINE-1 Methylation Level

Details of long interspersed nucleotide element 1 (LINE-1) methylation assay have been previously described [24], and we measured LINE-1 methylation levels in tumor DNA from over 1200 colorectal cancer cases in the cohorts [25]. Briefly, DNA was extracted from formalin-fixed paraffin-embedded tumor tissue sections. Bisulfite treatment of DNA, polymerase chain reaction (PCR), and a pyrosequencing assay were performed to measure "100 * C/(C + T)" (at each CpG site) where C indicates the amount of cytosine and T indicates that of thymine after bisulfite conversion of unmethylated cytosine to uracil (that was changed to thymine during subsequent PCR). LINE-1 methylation in each tumor was calculated as an average of 100 * C/(C + T) at four CpG sites, and treated as a continuous variable.

4. Supplementary Tables

Table S2. Age-Standardized Characteristics for Study Participants in the NHS (1980–2012) and the HPFS (1986–2012).

Characteristics	NHS (Women)	HPFS (Men)
Total person years	1,344,287	544,680
Age, years (mean, SD)	60.3 (11.3)	63.72 (11.2)
Alcohol, gram/day (median, IQR)	2.0 (0.2, 8.0)	6.1 (1.1, 15.2)
Ever smoking, %	56.1	51.6
Body mass index, kg/m^2		
<25, %	59.3	46.8
25–30, %	28.2	44.4
≧30, %	12.5	8.9
Physical activity, METS-hour/week (mean, SD)	16.1 (17.9)	25.7 (23.0)
Family history of colorectal cancer, %	16.6	13.6
Regular aspirin use, %	40.3	46.9
History of colon polyps, %	0.6	7.4
Multi-vitamin use, %	48.5	46.7
Folate, μg/day (mean, SD)	424.3 (212.3)	543.09 (253.9)
Calcium, mg/day (mean, SD)	929.4 (357.1)	933.8 (373.2)
Red meat intake, servings/day (mean, SD)	0.9 (0.5)	0.9 (0.6)

All variables except age are age adjusted. Means (SD) or medians (IQR) are presented for continuous variables; percentages are presented for categorical variables. Cumulative average values throughout follow-up are presented for alcohol, folate, calcium, red meat intake, physical activity and body mass index. Updated values are presented for other variables. Abbreviations: HPFS, Health Professionals Follow-up Study; IQR, interquartile range; METS, metabolic equivalent task score; NHS, Nurses' Health Study; NSAID, nonsteroidal anti-inflammatory drug; SD, standard deviation.

Table S3. Model Testing for the Association of Alcohol Intake (≤15 g/day Versus 0g/day) with Colorectal Cancer Incidence, Based on Main Model in Three Functions and Three Settings.

Knots	Model Assessment	NHS	HPFS	Combined
K = 2	*p*-value			
	Overall	0.37	0.54	0.29
	Heterogeneity	-	-	-
	BIC	11634	7784	20436
	AIC	11586	7739	20386
K = 3	*p*-value			
	Overall	0.51	0.62	0.28
	Heterogeneity	-	-	-
	Nonlinearity	-	-	-
	BIC	11660	7804	20464
	AIC	11588	7736	20389
K = 4	*p*-value			
	Overall	0.47	0.78	0.34
	Heterogeneity	-	-	-
	Nonlinearity	-	-	-
	BIC	11686	7830	20492
	AIC	11589	7741	20393

All *p*-values reported above are two sided hypothesis testing:
(i) H_0: the intercept and all the coefficients in $g(\phi,Z)$ are zero (the overall test);
(ii) H_0: all the coefficients in $g(\phi,Z)$ except the intercept are zero (test for heterogeneity);
(iii) H_0: all the coefficients of the nonlinear terms in $g(\phi,Z)$ are zero (test for nonlinearity).
Abbreviations: AIC, Akaike's information criterion; BIC, Bayesian information criterion; HPFS, Health Professionals Follow-up Study; LINE-1, long interspersed nucleotide element-1; NHS, Nurses' Health Study.

Table S4. Hazard Ratio for Alcohol Intake (≤15 g/day Versus 0g/day) Modeled as Three Functions of LINE-1 Marker Value in Three Cohort Settings, Based on the Main Model.

Cohort	LINE-1 Methylation Level	Hazard Ratio with 95% Confidence Interval					
		Linear Function (K=2)		Restricted Cubic Spline (K = 3 knots)		Restricted Cubic Spline (K = 4 knots)	
Combined	30	1.35	(0.86, 2.11)	1.77	(0.87, 3.58)	1.47	(0.61, 3.53)
	40	1.25	(0.90, 1.74)	1.48	(0.92, 2.37)	1.32	(0.75, 2.33)
	50	1.16	(0.93, 1.44)	1.28	(0.95, 1.73)	1.13	(0.72, 1.79)
	60	1.07	(0.94, 1.23)	1.20	(0.92, 1.56)	1.03	(0.65, 1.63)
	70	0.99	(0.86, 1.15)	1.21	(0.79, 1.85)	1.33	(0.81, 2.18)
	80	0.92	(0.72, 1.17)	1.27	(0.64, 2.53)	2.37	(0.38, 14.9)
HPFS	30	1.30	(0.61, 2.77)	1.71	(0.54, 5.40)	1.63	(0.39, 6.87)
	40	1.23	(0.71, 2.14)	1.44	(0.68, 3.08)	1.41	(0.57, 3.46)
	50	1.16	(0.81, 1.67)	1.27	(0.80, 2.02)	1.24	(0.64, 2.41)
	60	1.10	(0.87, 1.38)	1.21	(0.82, 1.76)	1.16	(0.65, 2.08)
	70	1.03	(0.80, 1.34)	1.24	(0.67, 2.27)	1.27	(0.51, 3.19)
	80	0.98	(0.65, 1.48)	1.32	(0.48, 3.60)	1.58	(0.06, 41.7)
NHS	30	1.45	(0.83, 2.55)	1.69	(0.69, 4.15)	1.24	(0.41, 3.78)
	40	1.31	(0.87, 1.99)	1.45	(0.79, 2.65)	1.19	(0.57, 2.49)
	50	1.19	(0.90, 1.56)	1.26	(0.86, 1.87)	1.00	(0.53, 1.87)
	60	1.08	(0.91, 1.28)	1.15	(0.80, 1.65)	0.84	(0.42, 1.70)
	70	0.97	(0.81, 1.17)	1.09	(0.60, 1.98)	1.17	(0.67, 2.03)
	80	0.88	(0.65, 1.19)	1.06	(0.41, 2.75)	2.63	(0.37, 18.4)

Abbreviations: HPFS, Health Professionals Follow-up Study; LINE-1, long interspersed nucleotide element-1; NHS, Nurses' Health Study.

5. Supplementary Figure

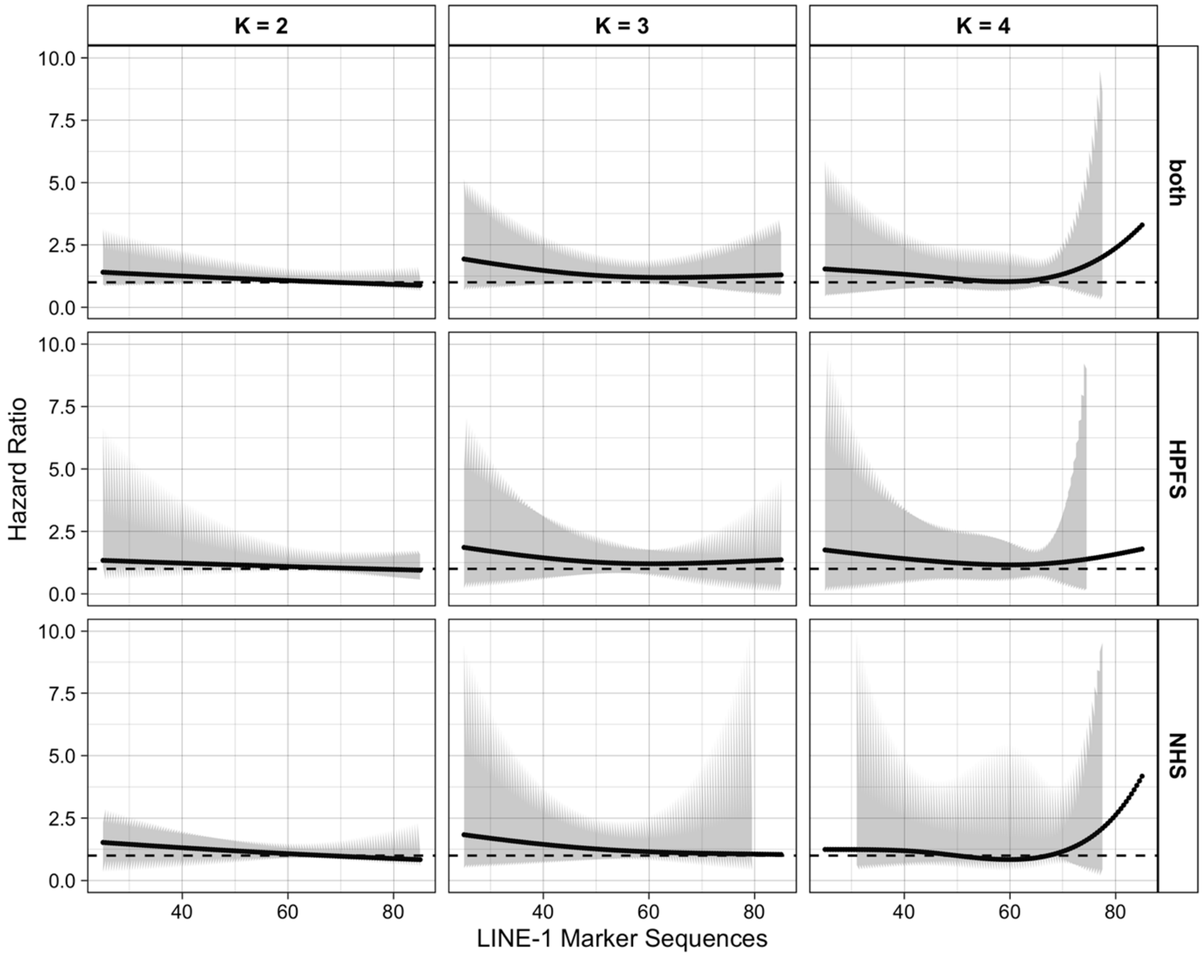

Figure S1. Heterogeneous Effect of Cumulative Categorical Alcohol Intake (≤15 g/day Versus <0g /day) on Continuous Subtypes of Colorectal Cancer; the 3 × 3 plot panel illustrates the combination of three choices of the knot number in $g(\phi, Z)$ and three cohort settings. Abbreviations: HPFS, Health Professionals Follow-up Study; LINE-1, long interspersed nucleotide element-1; NHS, Nurses' Health Study.
